# Anion exchange membranes containing no β-hydrogen atoms on ammonium groups: synthesis, properties, and alkaline stability†

**DOI:** 10.1039/d0ra09308d

**Published:** 2021-01-04

**Authors:** Daniel Koronka, Kenji Miyatake

**Affiliations:** Interdisciplinary Graduate School of Medicine and Engineering, University of Yamanashi 4 Takeda Kofu 400-8510 Japan; Fuel Cell Nanomaterials Center, University of Yamanashi 4 Takeda Kofu 400-8510 Japan miyatake@yamanashi.ac.jp; Clean Energy Research Center, University of Yamanashi 4 Takeda Kofu 400-8510 Japan; Department of Applied Chemistry, Waseda University Tokyo 169-8555 Japan kmiyatake@aoni.waseda.jp

## Abstract

Novel anion conductive polymer membranes have been designed and synthesized to investigate whether the absence of β-hydrogen atoms of ammonium groups affects the membranes' properties and chemical stability. The hydrophilic monomer, 2,2-bis(4-chlorobenzyl)-2-phenyl-ethylamine (3), was obtained *via* a two-step reaction with an overall yield of 98% under mild reaction conditions. Ni(0)-promoted copolymerization of 3 with 2,2-bis(4-chlorophenyl)hexafluoropropane (1) afforded high molecular weight copolymers (*M*_n_ = 12.8–19.6 kDa, *M*_w_ = 82.1–224.6 kDa). After quaternization with iodomethane, QBAF-BS polymers formed bendable, robust membranes from solution casting. The ion exchange capacity (IEC) of the membranes ranged from 1.50 to 2.44 mequiv. g^−1^. The membranes exhibited high hydroxide ion conductivity in water (up to 191 mS cm^−1^ at 80 °C for IEC = 2.25 mequiv. g^−1^), suggesting that the newly designed hydrophilic structure was effective in improving the ion conductivity. Based on small-angle X-ray scattering (SAXS) analyses and transmission electron microscopy (TEM) images, all membranes featured nano-phase separated morphology with a large dependence on the copolymer composition. The strain properties were improved on increasing the content of the hydrophilic component up to IEC = 2.25 mequiv. g^−1^, above which the strain became smaller due to the larger water absorption. The membranes were not stable under harsh alkaline conditions (in 8 M KOH at 80 °C) gradually losing the hydroxide ion conductivity. Compared to our previous AEMs which contained typical aliphatic ammonium groups, the lack of β-hydrogen atoms did not practically improve the alkaline stability of AEMs possibly due to the main chain degradation but contributed to higher ion conductivity.

## Abstract 2

A novel hydrophilic structure with no β-hydrogen atoms on the quaternary ammonium functionality has been developed for anion exchange membranes. The membranes featured high hydroxide conductivity and alkaline stability under harsh conditions.

## Graphical abstract

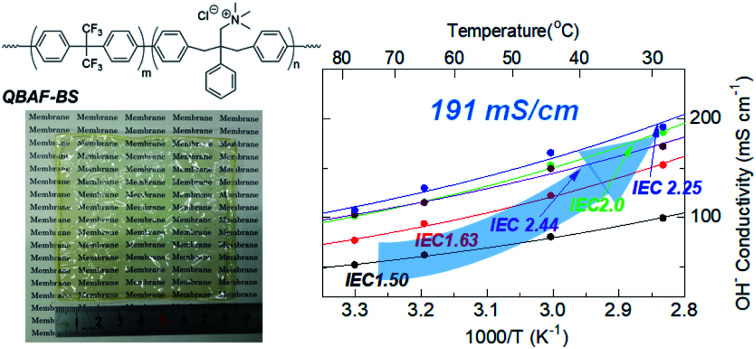

## 1. Introduction

Polymer electrolyte fuel cells (PEFCs) have been regarded as a viable solution for green, efficient and sustainable energy conversion devices that could replace old, environmentally harmful technologies using fossil fuels. The most prominent and widely researched type of PEFC is the so-called proton exchange membrane fuel cell (PEMFC). They are, however, rather costly which hinders the widespread dissemination of PEMFCs. In recent years, anion exchange membrane fuel cells (AEMFCs) have emerged as an alternative option, which are potentially advantageous over PEMFCs. In contrast to PEMFCs, the operating conditions for AEMFCs are not acidic but basic, therefore, inexpensive components can be used in the cells such as catalysts, bipolar plates, and even membranes.^1–4^ Currently, technical issues associated with AEMFCs are the membranes that are prone to hydroxide ion-induced degradation under the basic conditions. One of the dominant degradation pathways is the Hofmann elimination in which hydroxide ions attack hydrogen atoms at β-position to the quaternary nitrogen, resulting in the formation of an alkene and a neutral tertiary amine.^5^

Several synthetic methods have been developed and investigated to overcome this issue including but not limited to comb-shaped polymers,^6^ pendant ammonium head groups strategy,^7^ polymer blends,^8^ cross-linking,^9^ grafting,^10^ and ammonium groups modifications.^11^ One of the promising approaches to improve the alkaline stability of AEMs is to design structures that are devoid of or have a minimal number of β-hydrogen atoms to suppress Hofmann degradation.^12–15^ A good example is poly(aryl imidazoliums) (PBIs) containing quaternary imidazolium groups in the main chain that showed outstanding stability in 10 M KOH at 100 °C for as long as 168 h while having 10 mS cm^−1^ of Cl^−^ conductivity at 25 °C in water.^16,17^ For examples beyond other PBIs,^18–20^ great alkaline stability was reported for blend membrane of poly(vinyl alcohol) (PVA) and poly(diallyldimethylammonium chloride) (PDDA) to survive in 8 M KOH up to 360 h with a hydroxide ion conductivity of 25 mS cm^−1^ in water at 25 °C.^21,22^ Benzyl halides in the polymer backbone or side chains when reacted with trimethylamine (TMA) or hexamethylenetetramine (HMTA) formed structures that contained no or less β-hydrogen atoms to the quaternary nitrogen.^23,24^ In these cases, however, a different degradation mode became dominant where the hydroxide ion attacked at the α-carbon leading to deamination. In most cases, however, synthesis of polymers with no β-hydrogen atoms contained complex processes and therefore, a potential industrial scale-up would be costly.

Previously, we developed partially fluorinated anion conductive polymers that exhibited high hydroxide ion conductivity and mechanical strength. Copolymer membranes (QPAF-4) containing perfluoroalkylene and pendant trimethylalkylammonium groups exhibited reasonable hydroxide ion conductivity up to 76 mS cm^−1^ at 80 °C in water. QPAF-4 membrane was stable in 1 M KOH at 80 °C for 1000 h without losing conductivity, however, unstable under severer conditions in 8 M KOH.^25^ More recently, hexafluoroisopropylidene groups were investigated as hydrophobic components to repress water swelling and consequently improve hydroxide ion conductivity (up to 134 mS cm^−1^ at 80 °C) compared to that of QPAF-4.^26^ Since these polymers shared the same pendant trimethylalkylammonium groups, alkaline (in)stability was similar.

In the present paper, we designed a novel hydrophilic structure with no β-hydrogen atoms at the ammonium groups for our partially fluorinated polymers. Unlike most of the previous polymers, precursor monomer and polymers, and the resulting quaternized copolymers could be synthesized in facile processes. Synthesis, structure, and chemical and physical properties of the copolymer membranes are reported to reveal the effect of the hydrophilic component.

## 2. Experimental section

### 2.1. Materials

2,2-Bis(4-aminophenyl)hexafluoropropane (>98.0%), 4-chlorobenzyl bromide (>95.0%), phenylacetonitrile (>98.0%), sodium hydride (60% dispersion in paraffin liquid), tetrahydrofuran anhydrous (stabilized with 2,6-di-*tert*-butyl-4-methylphenol, >99.5%), diethyl ether anhydrous (stabilized with 2,6-di-*tert*-butyl-4-methylphenol, >99.5%), cesium carbonate (>98.0%), iodomethane (>98.5%), and 2,2′-bipyridyl (bpy, >99%) were purchased from Tokyo Chemical Industry Co., Ltd, and used as received. Hydrochloric acid (35.0–37.0%), potassium hydroxide (>86%), sodium chloride (99%), sodium carbonate (>99.8%), sodium thiosulfate (>99.0%), sodium nitrate (>99%), sodium hydrogen carbonate (>99.5%), sodium nitrite (>98.5%), silver nitrate aq. (0.01 M), dimethyl sulfoxide (DMSO, >99%), bis(1,5-cyclooctadiene)nickel(0) (Ni(COD)_2_, 95%), *N*,*N*-dimethylacetamide (>99%), hexane (>96%), dichloromethane (DCM, >99.5%), silica gel N60 (spherical, neutral, 100–210 μm), sodium sulfate (>98.5%), magnesium sulfate, anhydrous (>95.0%), diethyl ether (>99.0%) and lithium aluminum hydride (LAH, >92.0%) were purchased from Kanto Chemical Co., Inc., and used as received. Dimethylsulfoxide-*d*_6_ with 0.03% TMS (DMSO-*d*_6_, Acros Organics) and chloroform-*d*_1_ (CDCl_3_, Acros Organics) were used as received.

### 2.2. Synthesis of monomers

#### 2,2-Bis(4-chlorophenyl)-hexafluoropropane (1)

2,2-Bis(4-aminophenyl)-hexafluoropropane (1.0 g, 3.0 mmol) was mixed with conc. HCl (10 mL) in a 100 mL round-bottom flask while cooled with an ice-water bath. To this suspension, a solution of sodium nitrite (0.52 g, 7.5 mmol) in water (10 mL) was added dropwise over a period of 30 min. After the addition, the mixture was allowed to stir at 0–5 °C for 30 min. To the reaction mixture a pre-cooled solution of copper(i) chloride (1.512 g, 15.3 mmol) in conc. HCl (10 mL) was added dropwise to maintain the reaction temperature below 5 °C. Then, the mixture was stirred at r.t. for 2 h. After the reaction, the mixture was extracted with three portions of DCM (20 mL). The organic layers were collected, washed with brine and dried over sodium sulfate. The mixture was evaporated under reduced pressure to obtain crude product as an orange viscous liquid. The crude product was purified *via* flash chromatography (eluent: hexane) to afford pure 1 as a white solid (0.783 g, 65%). ^1^H NMR (500 MHz, CDCl_3_): *δ* 7.30 (d, *J* = 9.2 Hz, 4H), 7.36 (d, *J* = 8.1 Hz, 4H).

#### 2,2-Bis(4-chlorobenzyl)-2-phenylacetonitrile (2)

Anhydrous THF (40 mL) was added to a three-neck round-bottom flask under nitrogen atmosphere while cooled with an ice-water bath. Sodium hydride (2.0 g, 50 mmol) followed by phenyl acetonitrile (1.17 g, 10 mmol) was added with vigorous stirring. To the mixture, 4-chlorobenzyl bromide (5.13 g, 25 mmol) was added. The mixture was stirred at r.t. under nitrogen atmosphere for 24 h. Then, the mixture was cooled again with an ice-water bath, and water (20 mL) was added dropwise to quench the reaction. The mixture was extracted with three portions of diethyl ether (20 mL). The organic layers were collected, washed with brine and dried over sodium sulfate. The mixture was evaporated under reduced pressure to obtain crude product as an orange viscous liquid. The crude product was triturated thoroughly with hexane to afford pure 2 as a white solid (1.81 g, 98%). ^1^H NMR (500 MHz, CDCl_3_): *δ* 3.27 (s, 4H), 7.13 (t, *J* = 7.5 Hz, 4H), 7.8 (d, *J* = 8.0 Hz, 4H).

#### 2,2-Bis(4-chlorobenzyl)-2-phenyl-ethylamine (3)

A three-neck round-bottom flask equipped with a magnetic stirring bar, a nitrogen inlet and outlet, and a reflux condenser was charged with anhydrous diethyl ether (30 mL). While cooled with an ice-water bath, LAH (1.05 g, 27.6 mmol) followed by a solution of 2 (2.0 g, 5.43 mmol) in anhydrous diethyl ether (20 mL) was added. The mixture was heated to reflux (40 °C) for 2 h. The mixture was cooled again with an ice-water bath, and the reaction was quenched by the dropwise addition of water (1.0 mL), 20 wt% sodium sulfate aqueous solution (1.0 mL), and water (3.0 mL). After stirring for 30 min to deactivate any unreacted LAH, anhydrous MgSO_4_ was added to the mixture and allowed to stir for another 30 min. The resulting white precipitate was removed by filtration through a celite plug. The removal of diethyl ether solvent under reduced pressure afforded pure 3 as a colorless oil (2.0 g, 99%), which solidified over extended storage below 10 °C to form a white crystalline powder. ^1^H NMR (500 MHz, CDCl_3_): *δ* 0.94 (s, 2H), 2.80 (s, 2H), 3.08 (s, 4H), 7.07 (t, *J* = 8.0 Hz, 4H), 7.19 (d, *J* = 8.0 Hz, 4H), 7.21–7.33 (m, 5H).

### 2.3. Polymerization

A three-neck round-bottom flask equipped with a magnetic stirring bar, a nitrogen inlet and outlet, and a reflux condenser was charged with 1 (336.5 mg, 0.90 mmol), 3 (311 mg, 0.83 mmol), bpy (680 mg, 4.36 mmol), and dry DMAc (6 mL) under nitrogen atmosphere. The mixture was heated to 80 °C with stirring, and Ni(COD)_2_ (1.200 g, 4.36 mmol) was added. The polymerization was carried out at 80 °C for 5 h. After cooled to r.t., the mixture was poured into a large excess of 1/1 (by volume) mixture of methanol and conc. hydrochloric acid. The resulting precipitate was washed with a mixture of methanol/hydrochloric acid, 1.0 M K_2_CO_3_ aq. twice, and ultra-pure water (18 MΩ). After drying in vacuum at 60 °C overnight, BAF-BS*x* (where *x* refers to the titrated IEC) polymer was obtained as a white fibrous solid (519.8 mg, 93%).

### 2.4. Quaternization and membrane casting

A typical procedure is as follows. BAF-BS (target IEC = 1.50 mequiv. g^−1^ for the resulting quaternized polymer) (450 mg, 0.65 mmol of the primary amino groups) was dissolved in DMSO (10 mL) in a round-bottom flask. To the solution, cesium carbonate (480 mg, 1.47 mmol) and iodomethane (3.14 g, 22.1 mmol) were added separately. The mixture was stirred under nitrogen atmosphere at 40 °C for 36 h, and then added dropwise into a mixture of water (200 mL), sodium chloride (50 g), sodium carbonate (5 g), and sodium thiosulfate (1.58 g) to precipitate quaternized QBAF-BS as a white solid in Cl^−^ ion form (addition of sodium carbonate and sodium thiosulfate was effective to avoid the formation of I_2_). The product was filtered, washed with ultra-pure water, dried at 60 °C overnight to yield a white solid (500 mg, 99%). To obtain the membranes, solution casting method was used with DMSO as solvent (5 mL). The solution was cast onto a flat glass surface at 55 °C overnight. The obtained colorless, bendable membranes were washed with water to remove any residual DMSO solvent.

### 2.5. Measurements

Mechanical analyses (dynamic mechanical analysis, stress *versus* strain tests) were carried out for the membranes in Cl^−^ forms to avoid the effect of atmospheric CO_2_. For alkaline stability tests and hydroxide ion conductivity measurements, membranes in OH^−^ ion forms were used. Ion exchange was carried out by immersing the membranes in 1.0 M KOH solution for 24 h at 40 °C. After the ion exchange reaction, the membranes were washed with ultra-pure water for 24 h at 40 °C. For TEM images, the membranes were ion-exchanged to PtCl_4_^2−^ ion forms. The properties measurements such as dynamic mechanical analysis (DMA), stress *versus* strain (SS) curves, hydroxide ion conductivity, water uptake, transmission electron microscopy (TEM), small-angle X-ray scattering (SAXS), and alkaline stability were carried out as described in our previous work.^19^

## 3. Results and discussion

### 3.1. Monomer syntheses

**Scheme 1:**
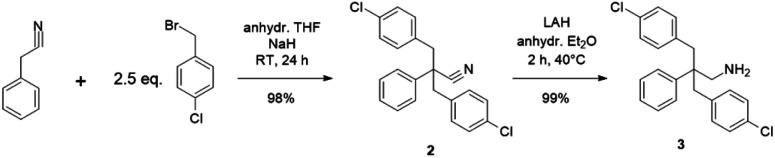
Synthesis of 2,2-bis(4-chlorobenzyl)-2-phenyl-ethylamine (3).

The hydrophobic monomer (1) was synthesized from 2,2-bis(4-aminophenyl)hexafluoropropane *via* Sandmeyer-reaction (Scheme S1†), of which chemical structure was confirmed by ^1^H, ^13^C, and ^19^F NMR spectra (Fig. S1–S3†). The hydrophilic monomer (3) was synthesized *via* a two-step reaction as shown in Scheme 1. The successful bis-4-chlorobenzylation of the phenylacetonitrile was suggested by the disappearance of the methylene proton peak (at 4.4 ppm, Fig. S4†) of the starting phenylacetonitrile in the ^1^H NMR spectrum (Fig. S5†). In addition, methylene peaks of 4-chlorobenzyl groups were observed at 3.3 ppm (Fig. S5†), which were at higher magnetic field than those of 4-benzylbromide at 3.8 ppm (Fig. S6†). In the ^13^C NMR spectrum, peaks were well-assigned to the structure (Fig. S7†). The second step was the reduction of the nitrile groups to primary amine groups using LAH. The reduction reaction proceeded well to provide the target compound without producing any by-products. The structure of the product was also confirmed by ^1^H and ^13^C NMR spectra (Fig. S5 and S7†). The new hydrophilic monomer (3) was obtained in >98% overall yield.

### 3.2. Polymerizations

#### Polymerization reactions

**Scheme 2:**
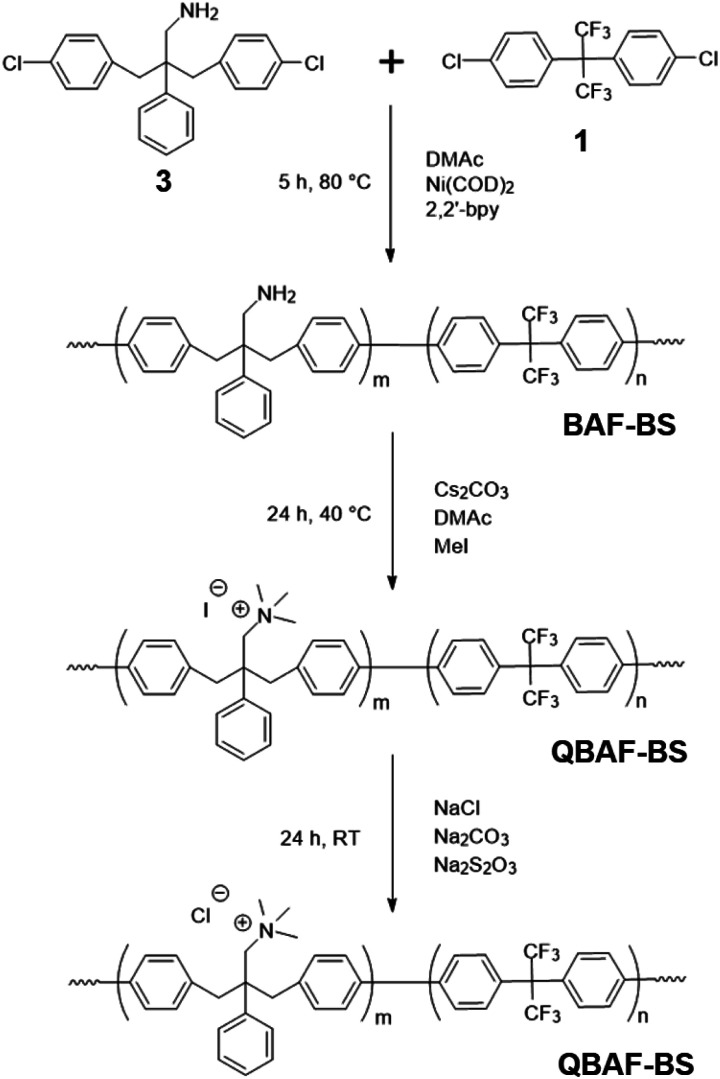
Synthesis of QBAF-BS copolymers.

**Fig. 1:**
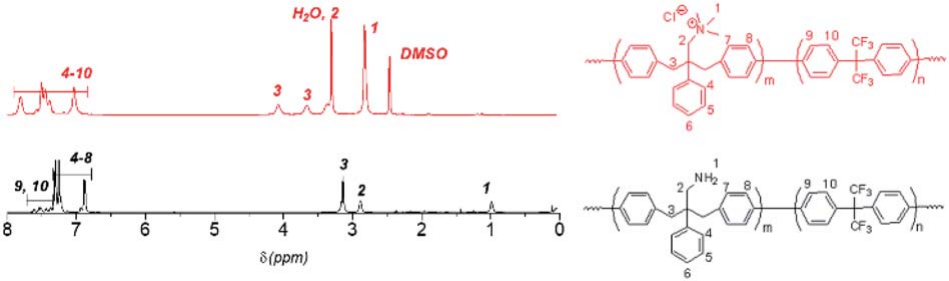
^1^H NMR spectra of QBAF-BS in DMSO-*d*_6_ (top) and BAF-BS in CDCl_3_ (bottom).

Ni(0)-promoted polycondensation of the monomers 1 and 3 was carried out under the conditions similar to those for our previously reported (Scheme 2).^27^ Comonomer feed ratios were controlled to obtain the final quaternized copolymers QBAF-BS with targeted ion exchange capacities (IEC) ranging from 1.50 to 2.50 mequiv. g^−1^. In all cases, the copolymers were obtained as white fibrous products in reasonably high yield, typically between 80–93% (Table 1). The precursor BAF-BS copolymers were readily soluble in organic solvents such as chloroform, DMSO or DMAc. The molecular weights estimated from GPC analyses were *M*_n_ = 12.8–19.6 kDa, *M*_w_ = 82.1–224.6 kDa with relatively high polydispersity (PDI = 5.3–12.2). Similarly high PDIs were obtained for our previous copolymers (BAF-QAF and QPAF-4) having tertiary amine groups under similar polymerization conditions, meaning less controllable Ni(0)-promoted reaction.^25,26^ In Fig. 1 the ^1^H NMR spectrum of BAF-BS2.00 copolymer as a typical example is shown, in which all peaks were well-assigned to the supposed structure suggesting successful copolymerization reaction. The copolymer compositions calculated from the peak integrals in the ^1^H NMR spectra were in good accordance with the feed comonomer composition within acceptable errors.

**Table 1:** Copolymer composition, target and obtained IECs, molecular weight and yield of QBAF-BS copolymers

No.	Composition (1 : 3)		Molecular weight^c^ (kDa)			IEC (mequiv. g^−1^)		Yield (%)
	Feed^a^	Obtained^b^	*M*_n_	*M*_w_	*M*_w_/*M*_n_	NMR^b^	Titration	
QBAF-BS1.50	1.07 : 1.00	1.17 : 1.00	12.8	84.5	6.6	1.39	1.50	93
QBAF-BS1.75	0.76 : 1.00	0.74 : 1.00	18.1	155.6	8.6	1.52	1.63	88
QBAF-BS2.00	0.51 : 1.00	0.51 : 1.00	16.4	181.9	11.1	1.99	2.02	82
QBAF-BS2.25	0.34 : 1.00	0.35 : 1.00	18.4	224.6	12.2	2.24	2.25	87
QBAF-BS2.50	0.19 : 1.00	0.22 : 1.00	19.6	104.0	5.3	2.32	2.44	80

a The molar ratio of the comonomers in the polymerization.

b Calculated from the peak integrals in the ^1^H NMR spectra.

c Measured for BAF-BS copolymers.

Methylation or quaternization reaction of the precursor BAF-BS copolymers was then carried out with large excess of iodomethane in DMSO solution in the presence of cesium carbonate to obtain QBAF-BS copolymers. The resulting products were white, fibrous solids, and fully soluble in DMSO and DMAc, and even soluble in methanol when the counter ions were chloride ions, but insoluble in less polar solvents such as chloroform. ^1^H NMR spectrum of QBAF-BS suggested a complete quaternization reaction by comparison with that of the precursor BAF-BS in Fig. 1. The quantitative methylation reaction was suggested by the disappearance of the peak of the primary amine groups at *ca.* 1.0 ppm and the appearance of the prominent peak of methyl protons on the quaternary ammonium groups at 2.8 ppm. The polymer backbone remained intact based on the fact that no shift was observed in the ^19^F NMR peaks (Fig. S8†). Ion exchange capacity (IEC) values calculated from the peak integrals in the ^1^H NMR spectra of QBAF-BS copolymers are summarized in Table 1. The titrated IEC (*via* Mohr-titration) and NMR-estimated IEC values were in fair agreement.

Casting from DMSO solution provided transparent, colorless and bendable membranes from all quaternized copolymers (Fig. S9†). Membrane flexibility seemed similar to that of our group's previously developed BAF-QAF^26^ regardless of its composition (the chemical structure of BAF-QAF polymer is shown in Fig. S10†).

### 3.3. Morphology

**Fig. 2:**
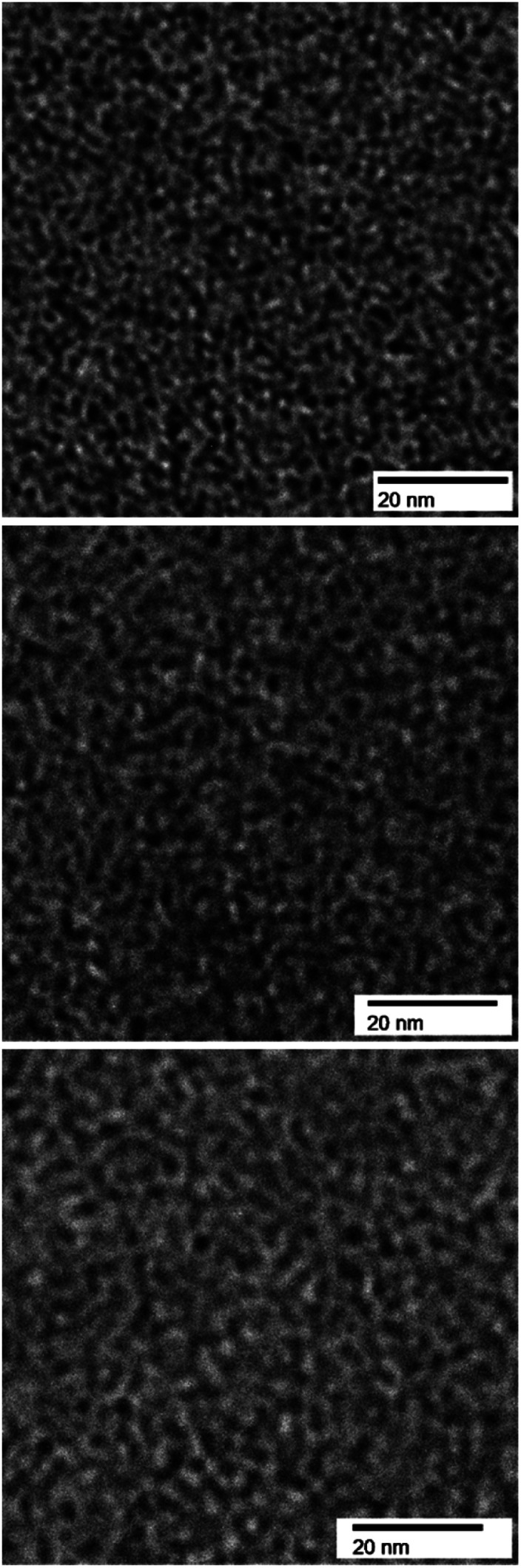
TEM images of QBAF-BS membranes with IEC = 1.50 mequiv. g^−1^ (top), IEC = 2.00 mequiv. g^−1^ (middle), and IEC = 2.44 mequiv. g^−1^ (bottom), all stained with tetrachloroplatinate ions.

Cross-sectional TEM images of QBAF-BS membranes with different IEC values, stained with tetrachloroplatinate ions are shown in Fig. 2. The membranes exhibited phase-separated morphology with the dark areas ascribed to hydrophilic and bright areas ascribed to hydrophobic domains, respectively. Both hydrophilic and hydrophobic domains were nearly spherical in shape, where the hydrophilic domains were similar in size, around 1.1–1.3 nm in diameter regardless of their IEC (1.3 ± 0.32 nm for 1.50 mequiv. g^−1^, 1.1 ± 0.21 nm for 2.00 mequiv. g^−1^ and 1.2 ± 0.24 nm for 2.44 mequiv. g^−1^). The average size of the hydrophobic clusters slightly increased as increasing IEC (1.22 ± 0.23 nm for 1.50 mequiv. g^−1^, 1.49 ± 0.20 nm for 2.00 mequiv. g^−1^ and 1.90 ± 0.31 nm for 2.44 mequiv. g^−1^). It is noted that the interface of the hydrophilic and hydrophobic domains was more distinct and sharper in contrast for lower IEC membranes. The result suggests that ionic groups were more incorporated into the hydrophobic domains in the higher IEC membranes probably because of the considerably larger composition of the hydrophilic components causing larger apparent size of the hydrophobic clusters.

**Fig. 3:**
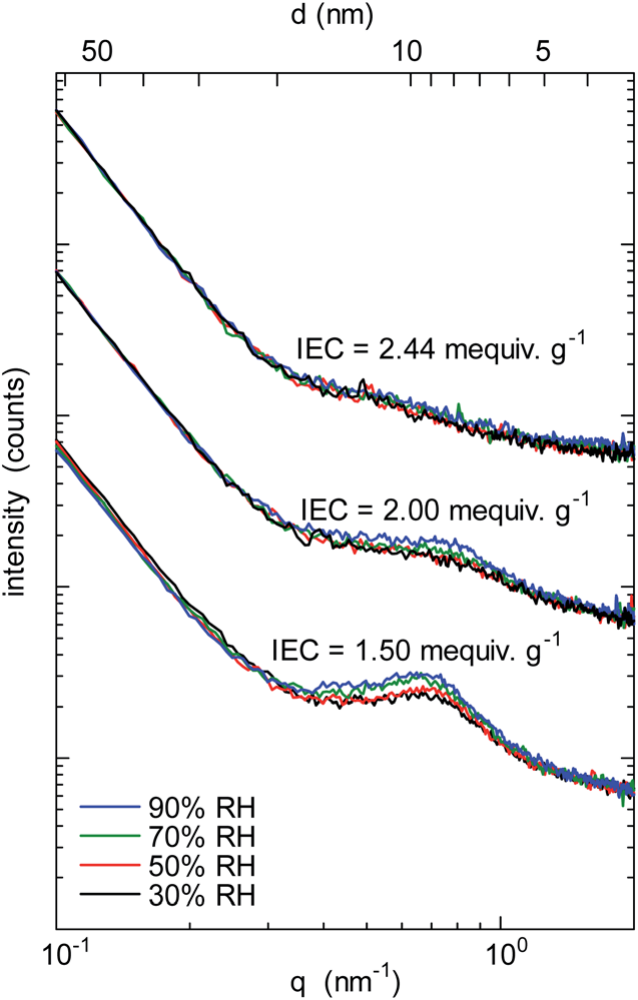
SAXS patterns of QBAF-BS membranes with IEC = 1.50 mequiv. g^−1^, IEC = 2.00 mequiv. g^−1^, and IEC = 2.44 mequiv. g^−1^ in Cl^−^ ion forms at 30%, 50%, 70% and 90% relative humidity.

To investigate the morphology under humidified conditions, small-angle X-ray scattering (SAXS) was measured at 40 °C and 30–90% RH (relative humidity) for the QBAF-BS membranes in Cl^−^ ion forms. Fig. 3 shows the scattering intensity as a function of the scattering vector (*q*) at different RH.

A distinct peak was observed at *q* = 0.66 nm^−1^ (or *d*-spacing = 9.5 nm) for the membrane with IEC = 1.50 mequiv. g^−1^. As the humidity increased, the peak slightly developed suggesting that the peak was associated with the hydrophilic clusters. A similar but much smaller peak was observed for higher IEC membranes with only minor dependence on the humidity. The peaks were roughly at *q* = 0.73 nm^−1^ (or *d*-spacing = 8.6 nm) and *q* = 0.57 nm^−1^ (or *d*-spacing = 11.0 nm) for IEC = 2.00 mequiv. g^−1^ and IEC = 2.44 mequiv. g^−1^, respectively. Similar to the TEM results, *d*-spacing slightly increased as increasing the IEC. Furthermore, the smaller peak supported the above-mentioned idea of less ordered (or more randomized) morphology for the higher IEC membranes.

### 3.4. Water uptake and ion conductivity

**Fig. 4:**
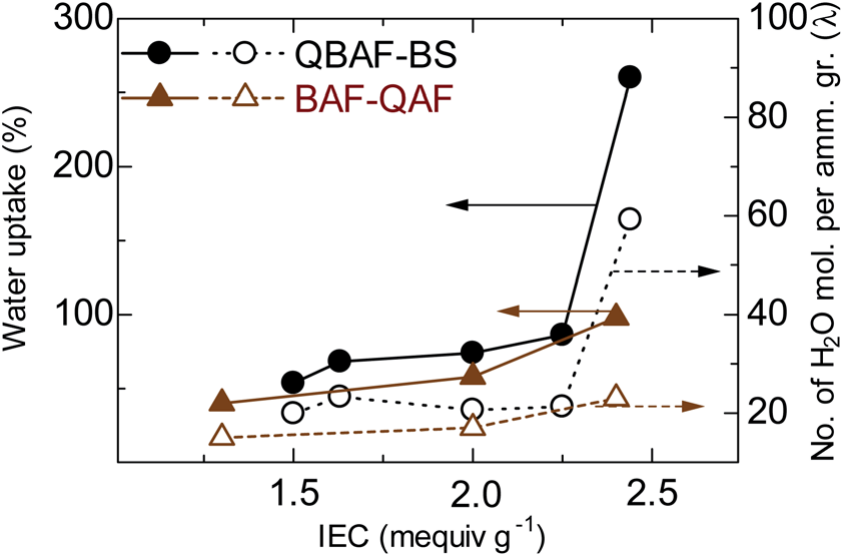
Water uptake (at r.t.) and *λ*-values of QBAF-BS and QBAF-BS membranes as a function of ion exchange capacity.

Fig. 4 shows water uptake (WU) and *λ*-value (number of absorbed water molecules per ammonium group) of QBAF-BS (and BAF-QAF for reference) membranes in OH^−^ ion forms at 30 °C as a function of the IEC. WU increased with increasing IEC to 86 wt% for QBAF-BS2.25, and jumped to 260 wt% for the highest IEC membrane QBAF-BS2.44. The *λ*-value remained nearly constant (*ca.* 20) up to IEC = 2.25 mequiv. g^−1^, and also jumped to 59 at the highest IEC membrane. The large increase in *λ* suggests that the water molecules were located in the hydrophobic domains as well as in the hydrophilic domains in the highest IEC membrane. This is probably not contradictory to the above-mentioned TEM images and SAXS data, where higher IEC membranes contained less ordered morphology. For comparison, BAF-QAF membranes sharing the same hydrophobic component showed water uptake increase from 40 wt% to 98 wt% and *λ* increase from 14 to 23 for 1.30 and 2.40 mequiv. g^−1^, respectively.^26^ A large jump of the water uptake and *λ* in BAF-QAF membrane at IEC = 2.40 mequiv. g^−1^, which was not observed for BAF-QAF membranes, might be ascribed to the ammonium groups attached closer to the polymer main chain. QBAF-BS membranes absorbed the same amount of water at 80 °C (Fig. S11†), suggesting good dimensional stability in hot water.

**Fig. 5:**
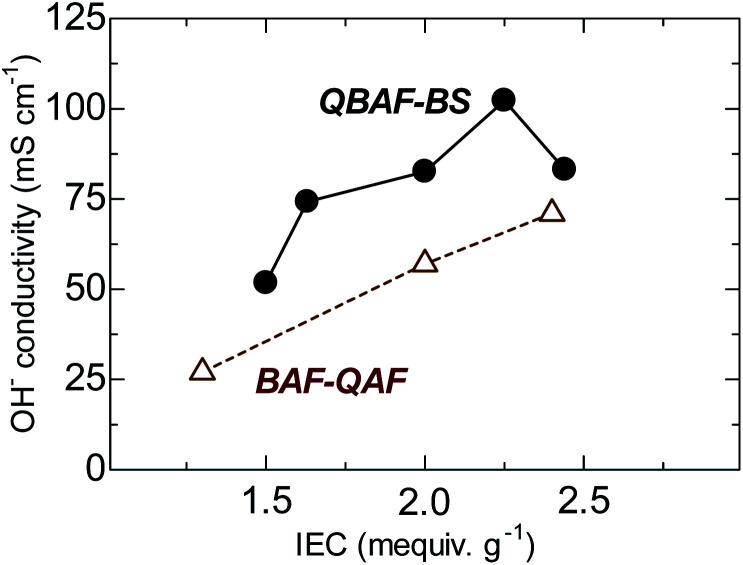
Hydroxide ion conductivity of QBAF-BS and BAF-QF membranes (in water, at 30 °C) as a function of ion exchange capacity.

The hydroxide ion conductivity of QBAF-BS membranes was measured in water at 30 °C and plotted as a function of IEC in Fig. 5. Unlike BAF-QAF membranes whose conductivity increased monotonously as increasing the IEC, QBAF-BS membranes exhibited a maximum conductivity of 102.3 mS cm^−1^ at IEC = 2.25 mequiv. g^−1^. The conductivity decreased to 83.2 mS cm^−1^ for the highest IEC membrane (2.44 mequiv. g^−1^). The excess water absorption at the highest IEC caused significant swelling of the membrane, resulting in the decrease in practical (volumetric) IEC and thus, the decrease in the conductivity. Similar behavior was observed with our previous membranes with large water absorbability.^25,28^ Compared to other quaternized polymer membranes devoid of β-hydrogen atoms (conductivity smaller than 100 mS cm^−1^ at 25 °C),^22,23^ QBAF-BS membranes showed higher hydroxide ion conductivity (102.3 mS cm^−1^ at 30 °C) for its relatively low IEC values.

**Fig. 6:**
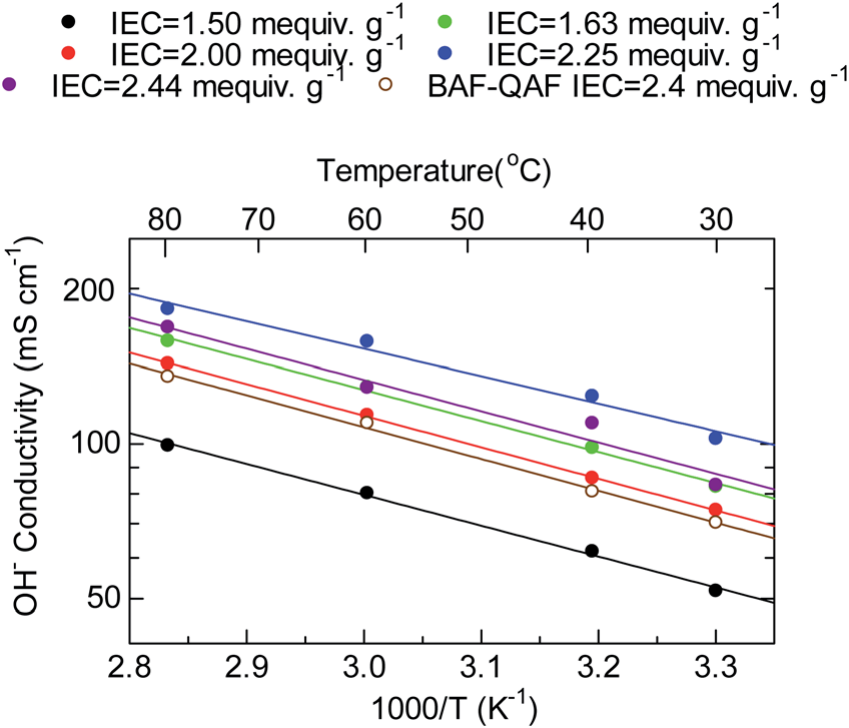
Temperature dependence of the hydroxide ion conductivity of QBAF-BS membranes.

Fig. 6 shows the temperature dependence of the hydroxide ion conductivity of the QBAF-BS membranes. All membranes followed Arrhenius-type temperature dependence from 30 to 80 °C with low apparent activation energies ranging between 10.2–11.7 kJ mol^−1^ suggesting a similar ion conduction mechanism for this series of membranes regardless of the IEC value. The *E*_a_ values were also similar to those of the BAF-QAF membranes (11–13 kJ mol^−1^), where migration of hydrated hydroxide ions *via* vehicular mechanism was suggested.^26^ Overall, QBAF-BS2.25 showed the best-balanced water uptake and conductivity properties within the 30 °C to 80 °C temperature range.

### 3.5. Mechanical properties

**Fig. 7:**
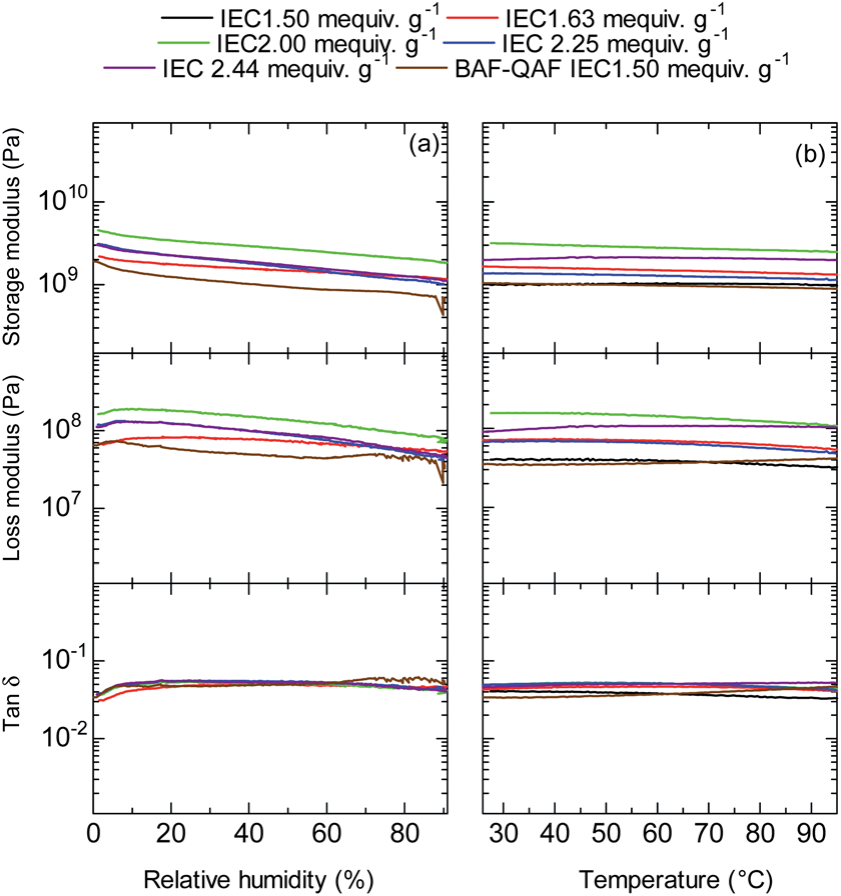
(a) Humidity (at 80 °C), and (b) temperature (at 60% RH) dependence of DMA curves of QBAF-BS membranes and BAF-QAF membrane for reference.

Dynamic mechanical analyses (DMA) of the QBAF-BS membranes were carried out in Cl^−^ ion forms. In the humidity dependence at 80 °C (Fig. 7a), storage (*E*′) and loss (*E*′′) moduli decreased gradually as increasing the humidity. No glass transition behaviour was observed for the three membranes in the tan *δ* (= *E*′/*E*′′) curves. Similarly, in the temperature dependence at 60% RH, the viscoelastic properties showed only minor changes without detectable peaks (Fig. 7b). The results are similar to those of the BAF-QAF membranes,^26^ indicating that the viscoelastic properties and their temperature/humidity dependences were mostly related with the hydrophobic components.

**Fig. 8:**
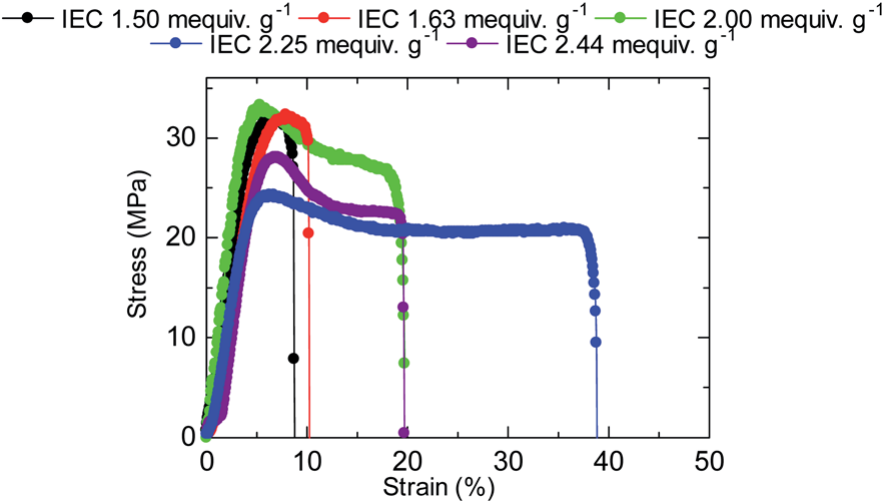
Stress *versus* strain curves of QBAF-BS membranes at 80 °C and 60% RH.

**Fig. 9:**
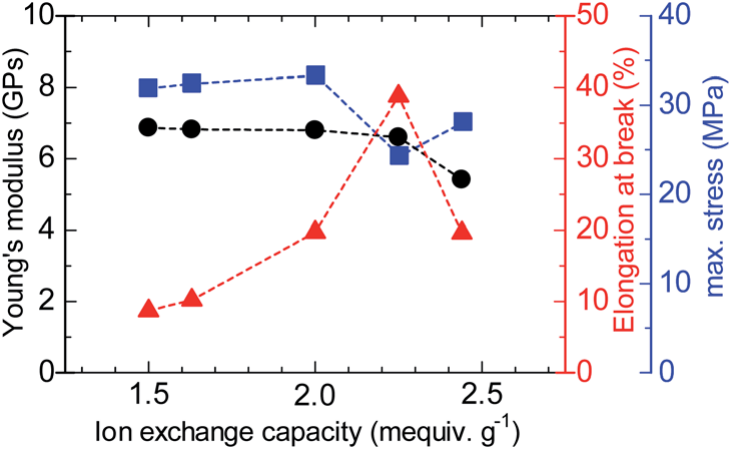
Mechanical properties (Young's modulus, elongation at break and maximum stress) of QBAF-BS membranes.

The stress *versus* strain curves of the QBAF-BS membranes were also measured in Cl^−^ ion forms at 80 °C and 60% RH (Fig. 8). The results are summarized in Fig. 9. Young's modulus slightly decreased with increasing IEC up to 2.25 mequiv. g^−1^ and dropped at 2.44 mequiv. g^−1^. The maximum stress at break showed similar IEC dependence. The IEC dependence of those properties is related with an increase in the absorbed water which acted as a plasticizer decreasing the membrane stiffness.^29^ In contrast, the maximum strain increased with increasing the IEC up to 2.25 mequiv. g^−1^, opposite IEC dependence from those of the Young's modulus and maximum stress. Although the membranes were bendable, the obtained maximum strains were relatively small with the highest for QBAF-BS2.25 (39%). Increase of the maximum strain with IEC supported the idea that the absorbed water increased the elasticity of the membranes. However, QBAF-BS2.44 showed a drop in the maximum strain presumably due to its much larger water absorbability (Fig. 4) and lower molecular weight (*M*_n_ = 19.6 kDa, *M*_w_ = 104 kDa) than those of QBAF-BS2.25 (*M*_n_ = 18.4 kDa, *M*_w_ = 224.6 kDa) (Table 1). Compared with BAF-QAF membranes, QBAF-BS membranes exhibited similar values and IEC dependence of the Young's modulus (BAF-QAF = 5.2–8.9 GPa). QBAF-BS showed larger strain with a maximum of 38.8% for IEC 2.25 mequiv. g^−1^ membrane (Fig. 8), compared to 13% for BAF-QAF (IEC 1.50 mequiv. g^−1^) membrane, which could be attributed to the hydrophilic component. The larger molecular weights (*M*_w_ = 84.5–224.3 kDa) of QBAF-BS membranes than those (*M*_w_ = 77–115 kDa) of BAF-QAF membranes were also accountable. It is concluded that the viscoelastic properties were dominated by the hydrophobic components while the strain properties were more related with the hydrophilic components, absorbed water, and molecular weight.

### 3.6. Alkaline stability

**Fig. 10:**
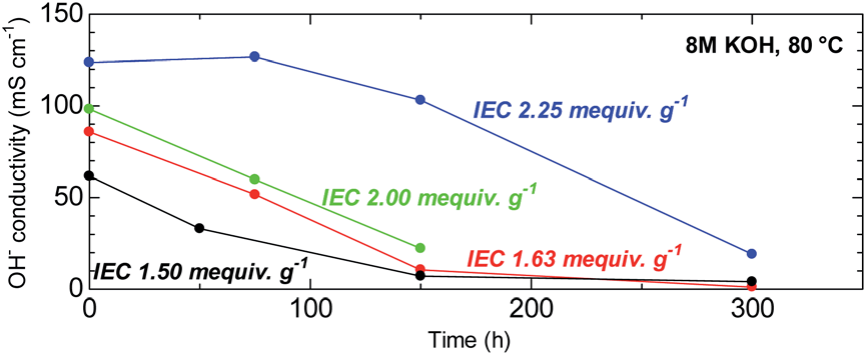
Time course of hydroxide ion conductivity (at 40 °C) of QBAF-BS membranes in 8 M KOH at 80 °C.

The ability to withstand harsh alkaline conditions is a crucial property of AEM membranes for alkaline fuel cell applications. The accelerated alkaline stability test using highly concentrated alkaline solution (8 M KOH) at elevated temperature (80 °C) used in this study was chosen not to reveal the expected life-span of this membrane in fuel cell conditions but to reveal the major degradation modes that are unique to this novel chemical structure. Furthermore, under the same conditions, BAF-QAF membrane showed major degradation.^26^ Thus, severe conditions served as a logical reference conditions for comparing QBAF-BS with BAF-QAF. Fig. 10 shows the remaining conductivity (in water at 40 °C) as a function of the testing time. Hydroxide ion conductivity of low IEC membranes (IEC = 1.50 and 1.63 mequiv. g^−1^) was lost after 300 h, with remaining conductivities of 4 mS cm^−1^ (6% of the initial conductivity) for IEC 1.50 mequiv. g^−1^ and 1 mS cm^−1^ (1% of the initial conductivity) for IEC 1.63 mequiv. g^−1^, respectively. The membrane with medium IEC (2.00 mequiv. g^−1^) also showed continuous degradation up to 150 h, at which the test was terminated with a remaining conductivity of 22 mS cm^−1^ (22% conductivity retention). The higher IEC (= 2.25 mequiv. g^−1^) membrane showed minor loss of the conductivity up to 150 h, however, the conductivity dropped after this time period to 19 mS cm^−1^ (85% loss of its initial conductivity) at 300 h. The highest IEC membrane (= 2.44 mequiv. g^−1^) showed severe swelling under the testing conditions causing mechanical failure that prohibited conductivity measurements. According to the estimated IEC values from the remaining conductivity values in Fig. 10, membranes with IEC < 2.00 mequiv. g^−1^ featured degradation with similar rate of the conductivity loss. A likely explanation for this result is in the increased activation energy of the hydroxide ion attack with higher *λ*-values at higher IECs.^30^

**Fig. 11:**
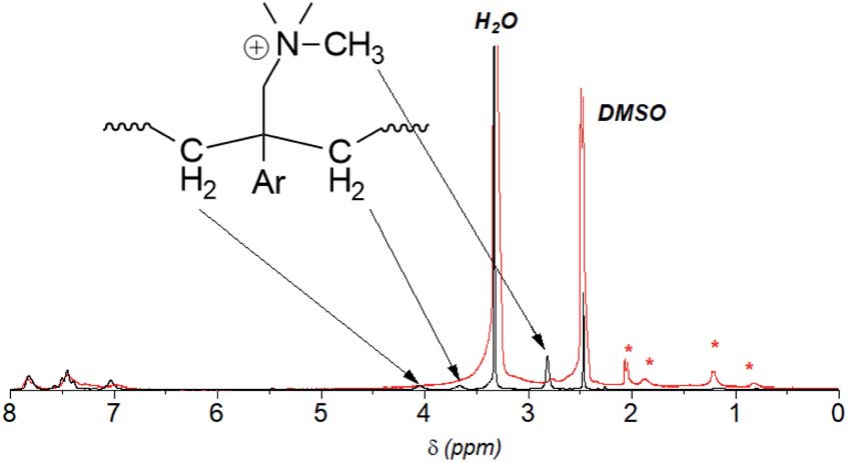
^1^H NMR spectrum of the pristine (black, 0 h) and post-test (red, 300 h) QBAF-BS1.50 membrane. The red asterisks (*) denote the new peaks that appeared after the alkaline stability test.

**Fig. 12:**
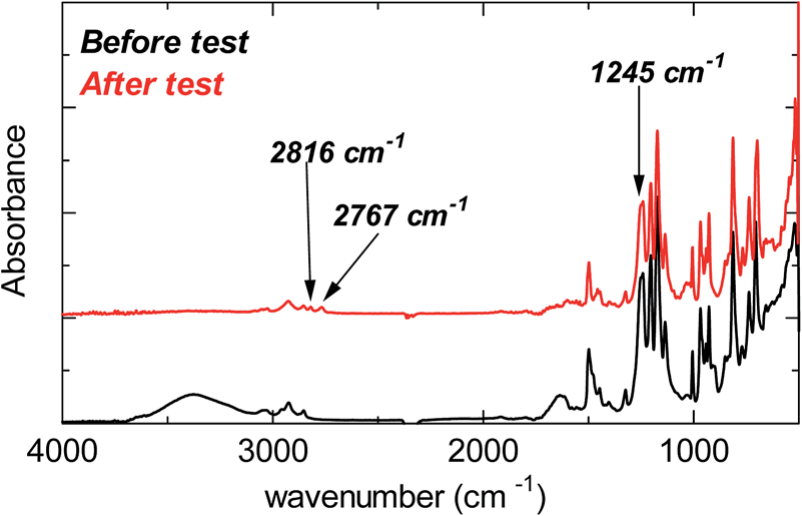
FTIR spectra of the pristine (black, 0 h) and post-test (red, 300 h) QBAF-BS1.50 membrane.

**Fig. 13:**
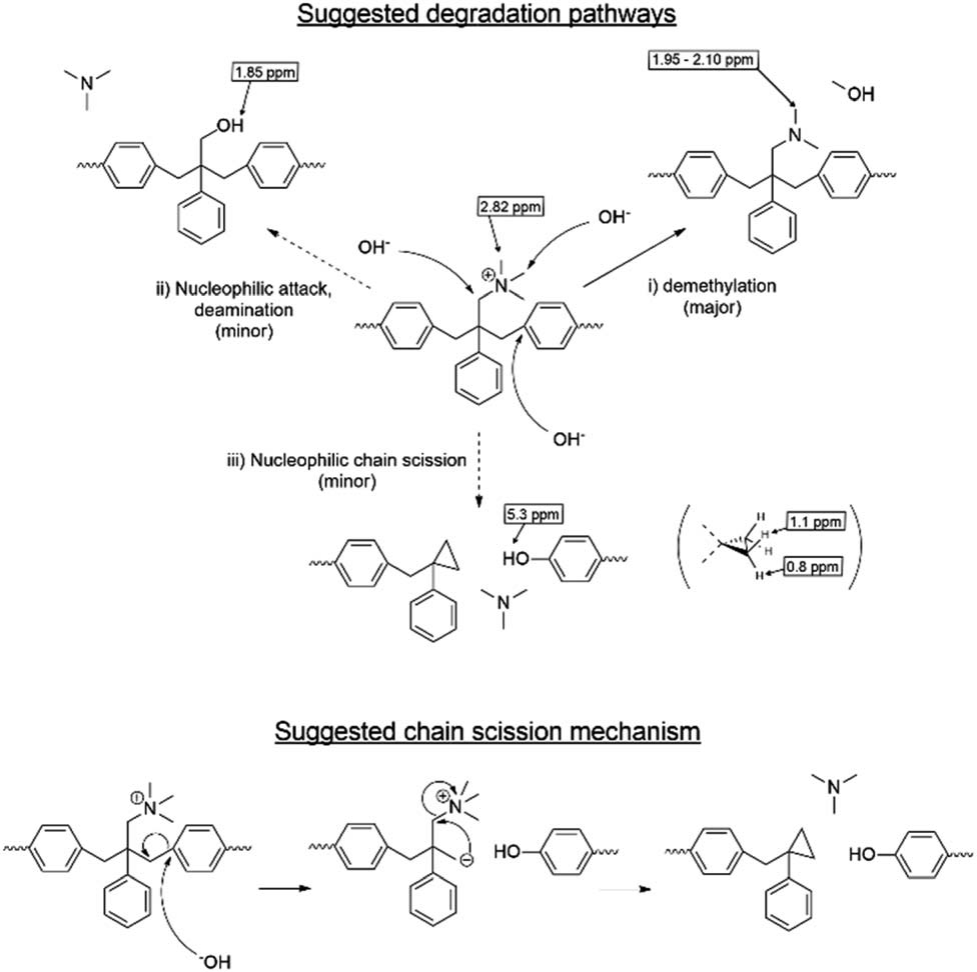
Possible hydroxide ion-induced degradation mechanism of QBAF-BS membranes and suggested chain cleavage mechanism based on the post-test NMR and FTIR spectra.

Despite the lack of hydrogen atoms at β-position to the quaternary nitrogen atoms, thereby eliminating the chance for Hofmann degradation, QBAF-BS membranes showed major loss of the hydroxide ion conductivity in 8 M KOH at 80 °C. To investigate the degradation mechanism of QBAF-BS membranes, ^1^H, ^19^F NMR and FTIR spectra were measured and compared with those of the pristine membranes. In Fig. 11, ^1^H NMR spectra of the QBAF-BS1.50 membrane is shown (see Fig. S12 and S13† for other membranes), where the prominent peak of the methyl groups attached to the quaternary trimethyl ammonium groups at 2.82–2.83 ppm disappeared after the stability test. A faint peak appeared at 5.3 ppm for all membranes (Fig. S14†). Furthermore, more prominent peaks appeared at 0.8, 1.1, 1.85 and around 2.0 ppm. ^19^F NMR spectra were nearly identical to those of the pristine samples (Fig. S15–S17†). In the FTIR spectra (Fig. 12, S18, and S19†), the peak at *ca.* 1245 cm^−1^ assignable to the stretching vibration of C–N^+^ bonds became smaller and new peaks assignable to the stretching vibration of CH_3_–N bonds appeared at 2767 cm^−1^ and 2816 cm^−1^ in the post-test samples. Based on the NMR and IR spectral data, suggested degradation mechanisms are summarized in Fig. 13. The disappearance of the quaternary ammonium peaks in the ^1^H NMR spectra together with the appearance of a prominent peak at 1.95–2.10 ppm assignable to methyl protons of tertiary amines suggests the demethylation of the trimethylammonium groups as a major degradation mode, leading to loss of IEC and conductivity (Fig. 13(i)). The changes in the FTIR spectra also support this idea. The appearance of the proton signals at 1.85 ppm in the ^1^H NMR spectrum could be the result of nucleophilic attack of the hydroxide ions on the methylene groups connecting with the quaternary ammonium groups to form alcohols (Fig. 13(ii)). In addition, the peaks at 5.3 ppm, and 0.8 and 1.1 ppm are indicative of phenolic protons and aryl cyclopropane groups, respectively, formed possibly *via* the mechanism shown in Fig. 13(iii). Loss of membrane flexibility observed for QBAF-BS would be a typical consequence of main chain degradation and a decrease in molecular weight (GPC measurement was unavailable for the quaternized polymers due to the strong interactions with the GPC columns). The post-test membranes were not strong enough for mechanical properties measurements. Since the ^19^F NMR spectra showed no changes, the hydrophobic component is believed to have remained intact through the alkaline stability test. Overall, the novel structure of QBAF-BS was effective in eliminating Hofmann-type degradation, which was the major degradation pathway for BAF-QAF polymers.^26^ However, the loss in ion conductivity and mechanical strength was consequence of the demethylation as major and nucleophilic main chain as minor degradation modes, respectively. In order to evaluate the fuel cell performance and long-term stability of the membranes, we tried preparation of catalyst coated membrane and fuel cell evaluation several times, only ended up in mechanical failure of the membranes. Higher molecular weight and/or smaller polydispersity would be needed for mechanically durable membranes. Since QBAF-BS is highly hydroxide ion conductive and soluble in methanol, fuel cell test will be conducted both as membrane and electrode ionomer (binder).

## 4. Conclusions

A series of novel anion conducting copolymers (QBAF-BS) containing hexafluoroisopropylidene and quaternary trimethylamine groups with no β-hydrogen atoms have been synthesized and characterized in detail. The copolymers showed excellent membrane forming capability resulting in freestanding, stiff but robust membranes with varying gravimetric ion exchange capacities ranging from 1.50 to 2.44 mequiv. g^−1^. Compared to our previous AEMs sharing the same hydrophobic component (BAF-QAF), lack of β-hydrogen atoms did not have much impact on water-absorbing capability at least up to IEC = 2.25 mequiv. g^−1^, above which swelling was significant. TEM and SAXS measurements suggested that QBAF-BS membranes showed a typical nano-phase separated morphology with similar hydrophilic and slightly increasing hydrophobic domains sizes with increasing IEC probably because of the stiff molecular structure of the hydrophilic component. Hydroxide ion conductivity in water displayed great IEC dependence with the highest conductivity (191 mS cm^−1^) for the membrane with IEC = 2.25 mequiv. g^−1^ at 80 °C, which was considerably higher than (134 mS cm^−1^) that of BAF-QAF membranes.^26^ Accelerated alkaline stability test in 8 M KOH at 80 °C induced significant loss of the ion conductivity within 300 h despite the lack of β-hydrogen atoms, which are well-known to experience Hofmann degradation in alkaline conditions. Post-alkaline stability analyses *via*^1^H, ^19^F, and FTIR spectra revealed other degradation modes, demethylation as major and nucleophilic main chain scission as minor, leading to the loss in ion conductivity and membrane flexibility.

## Conflicts of interest

There are no conflicts to declare.

## Supplementary Material

RA-011-D0RA09308D-s001

## References

[cit1] Setzler B. P., Zhuang Z., Wittkopf J. A., Yan Y. (2016). Nat. Nanotechnol..

[cit2] Peng X., Omasta T. J., Magliocca E., Wang L., Varcoe J. R., Mustain W. E. (2019). Angew. Chem., Int. Ed..

[cit3] Antolini E., Gonzalez E. R. (2010). J. Power Sources.

[cit4] Hossen M., Artyushkova K., Atanassov P., Serov A. (2018). J. Power Sources.

[cit5] Ghigo G., Cagnina S., Maranzana A., Tonachini G. (2010). J. Org. Chem..

[cit6] Li N., Leng Y., Hickner M. A., Wang C. Y. (2013). J. Am. Chem. Soc..

[cit7] Dang H. S., Jannasch P. (2015). Macromolecules.

[cit8] Li Z., He X., Jiang Z., Yin Y., Zhang B., He G., Tong Z., Wu H., Jiao K. (2017). Electrochim. Acta.

[cit9] Zhao Y., Yu H., Yang D., Li J., Shao Z., Yi B. (2013). J. Power Sources.

[cit10] Ponce-Gonzalez J., Whelligan D. K., Wang L., Bance-Soualhi R., Wang Y., Peng Y., Peng H., Apperley D. C., Sarode H. N., Pandey T. P., Divekar A. G., Seifert S., Herring A., Zhuang L., Varcoe J. R. (2016). Energy Environ. Sci..

[cit11] Mohanty A. D., Bae C. (2014). J. Mater. Chem. A.

[cit12] Mustain W. E., Chatenet M., Page M., Kim Y. S. (2020). Energy Environ. Sci..

[cit13] Liu L., Li Q., Dai J., Wang H., Jin B., Bai R. (2014). J. Membr. Sci..

[cit14] Lin B. C., Dong H. L., Li Y. Y., Si Z. H., Gu F. L., Yan F. (2013). Chem. Mater..

[cit15] Xue B., Dong X., Li Y., Zheng J., Li S., Zhang S. (2017). J. Membr. Sci..

[cit16] Fan J., Willdorf-Cohen S., Schibli E. M., Paula Z., Li W., Skalski T. J. G., Sergeenko A. T., Hohenadel A., Frisken B. J., Magliocca E., Mustain W. E., Diesendruck C. E., Dekel D. R., Holdcroft S. (2019). Nat. Commun..

[cit17] Fan J., Wright A. G., Britton B., Weissbach T., Skalski T. J., Ward J., Peckham T. J., Holdcroft S. (2017). ACS Macro Lett..

[cit18] Wright A. G., Fan J., Britton B., Weissbach T., Lee H.-F., Kitching E. A., Peckham T. J., Holdcroft S. (2016). Energy Environ. Sci..

[cit19] Thomas O. D., Soo K., Peckham T. J., Kulkarni M. P., Holdcroft S. (2012). J. Am. Chem. Soc..

[cit20] Ma H., Zhu H., Wang Z. (2019). J. Polym. Sci., Part A: Polym. Chem..

[cit21] Bauer B., Strathmann H., Effenberger F. (1990). Desalination.

[cit22] Qiao J., Zhang J., Zhang J. (2013). J. Power Sources.

[cit23] Vengatesan S., Santhi S., Sozhan G., Ravichandran S., Davidson D. J., Vasudevan S. (2015). RSC Adv..

[cit24] Koronka D., Mahmoud A. M. A., Miyatake K. (2019). J. Polym. Sci., Part A: Polym. Chem..

[cit25] Ono H., Kimura T., Takano A., Asazawa K., Miyake J., Inukai J., Miyatake K. (2017). J. Mater. Chem. A.

[cit26] Kimura T., Matusmoto A., Inukai J., Miyatake K. (2020). ACS Appl. Energy Mater..

[cit27] Koronka D., Matsumoto A., Otsuji K., Miyatake K. (2019). RSC Adv..

[cit28] Ono H., Miyake J., Shimada S., Uchida M., Miyatake K. (2015). J. Mater. Chem. A.

[cit29] Pan J., Lu S., Li Y., Huang A., Zhuang L., Lu J. (2010). Adv. Funct. Mater..

[cit30] Dekel D. R., Amar M., Willdorf S., Kosa M., Dhara S., Diesendruck C. E. (2017). Chem. Mater..

