# Supplementary material for: Anion exchange membranes containing no β-hydrogen atoms on ammonium groups: synthesis, properties, and alkaline stability
Source: RSC Adv. 2021 Jan 4;11(2):1030–8. doi: 10.1039/d0ra09308d (PMC8693587; doi:10.1039/d0ra09308d)
Supplement: RA-011-D0RA09308D-s001 [file RA-011-D0RA09308D-s001.pdf]

Electronic Supplementary Material (ESI) for RSC Advances.
This journal is © The Royal Society of Chemistry 2020

Supplementary information

Anion exchange membranes containing no β-hydrogen atoms on ammonium groups: synthesis, properties, and alkaline stability

Daniel Koronka[a] and Kenji Miyatake*[bcd]

[a]*Interdisciplinary Graduate School of Medicine, Engineering, and Agricultural Sciences, University of Yamanashi, 4 Takeda, Kofu, Yamanashi 400-8510, Japan*

[b]*Fuel Cell Nanomaterials Center, University of Yamanashi, 4 Takeda, Kofu 400-8510, Japan*

[c]*Clean Energy Research Center, University of Yamanashi, 4 Takeda, Kofu 400-8510, Japan*

[d]*Department of Applied Chemistry, Waseda University, Tokyo 169-8555, Japan*

Measurements. ^{1}H, ^{13}C, and ^{19}F NMR spectra were recorded in $CDCl_3$, or DMSO-d_6 containing tetramethylsilane as internal standard. The spectra recorded on a JEOL JNMECA500 spectrometer at room temperature. Molecular weight (M_n and M_w) of the BAF-BS polymers were estimated by gel permeation chromatography (GPC) using a Shodex KF-805L or SBSB-803 column with a Jasco 805 UV detector. Chloroform with 0.02 M added triethylamine was used as eluent and calibration was done using polystyrene standards. Dynamic mechanical analyses (DMA) (Storage modulus (E' (Pa)), loss modulus (E'' (Pa)) and tan δ (E''/E')) were measured on an ITK DVA-225 dynamic viscoelastic analyzer at either 60% relative humidity (RH) between room temperature and 95 °C at a heating rate of 1 °C min^{-1}, or as a function of RH at fixed 80 °C between 0% and 100% RH. Tensile strength of the membrane samples was determined at fixed 80 °C and 60% RH while stretching the membrane at a rate of 0.1 mm s^{-1}. Ion exchange capacity (IEC) of the quaternized membranes have been determined via Mohr titration method as reported in the literature.[1] Water uptake, ion conductivity, alkaline stability measurements were all carried out according to the literature.[1] For transmission electron microscopic (TEM) images, membrane samples (sized 5 mm by 5 mm) were ion exchanged to tetrachloroplatinate ions via submerging the samples into 0.5 M potassium tetrachloroplatinate (II) aqueous solution at 40 °C for 24 h. The ion exchange is followed by the washing of the membranes with deionized ultrapure (18 MΩ) water at 40 °C for 24 h and dried under high-vacuum overnight. The obtained dry membranes were embedded into an epoxy resin, cut to 50 nm thickness via a Leica microtome Ultracut UCT and placed onto a copper mesh. The images of the samples were taken using a Hitachi H-9500 transmission electron microscope.

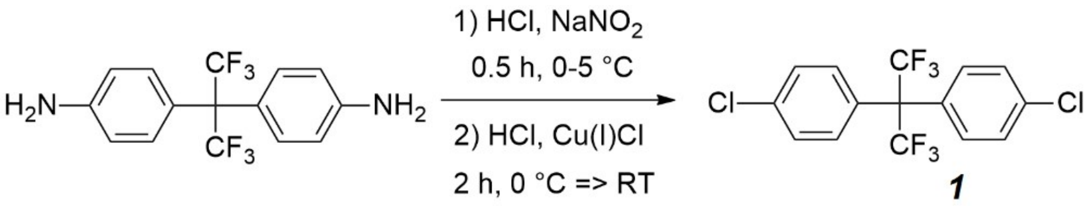

Scheme S1 Synthesis of 2,2-bis(4-chlorophenyl)-hexafluoropropane (**1**).

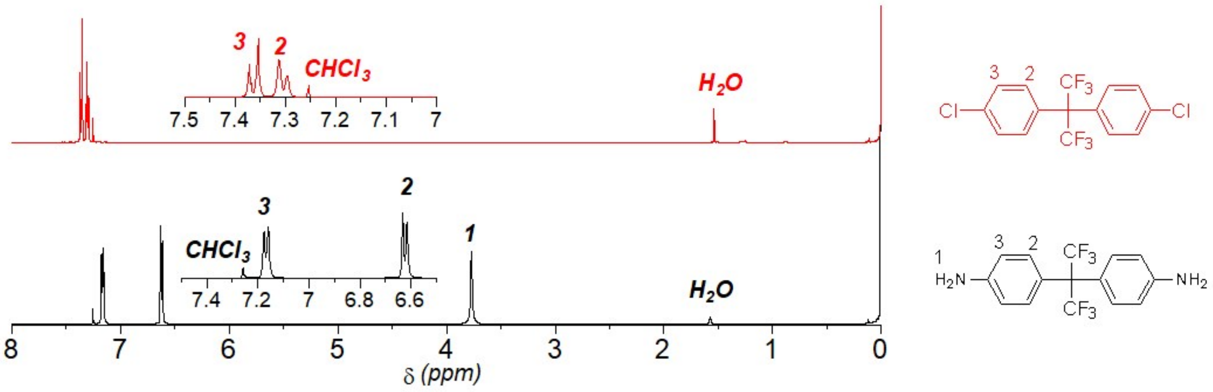

Fig. S1 ^{1}H NMR spectra of 2,2-bis(4-chlorophenyl)hexafluoropropane (**1**) (top) and 2,2-bis(4-aminophenyl)hexafluoropropane (bottom) in $CDCl_3$.

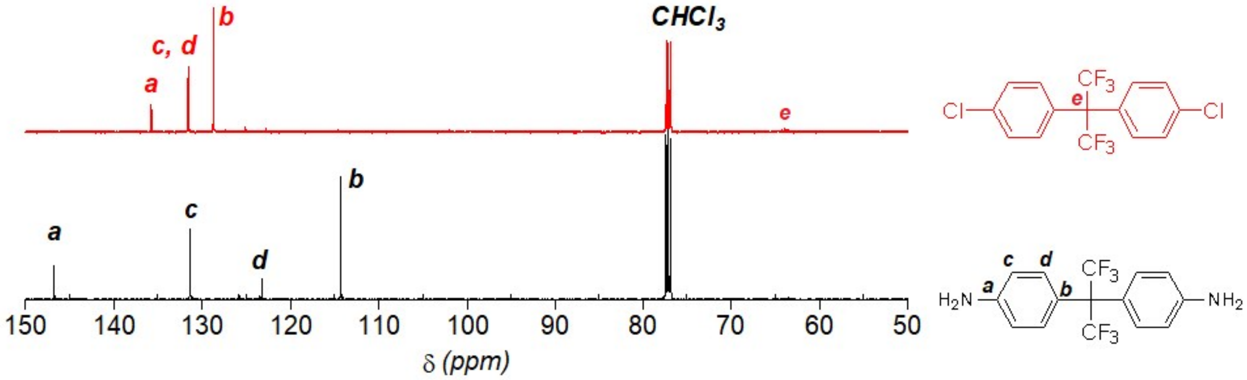

Fig. S2 ^{13}C NMR spectra of 2,2-bis(4-chlorophenyl)hexafluoropropane (**1**) (top) and 2,2-bis(4-aminophenyl)hexafluoropropane (bottom) in $CDCl_3$.

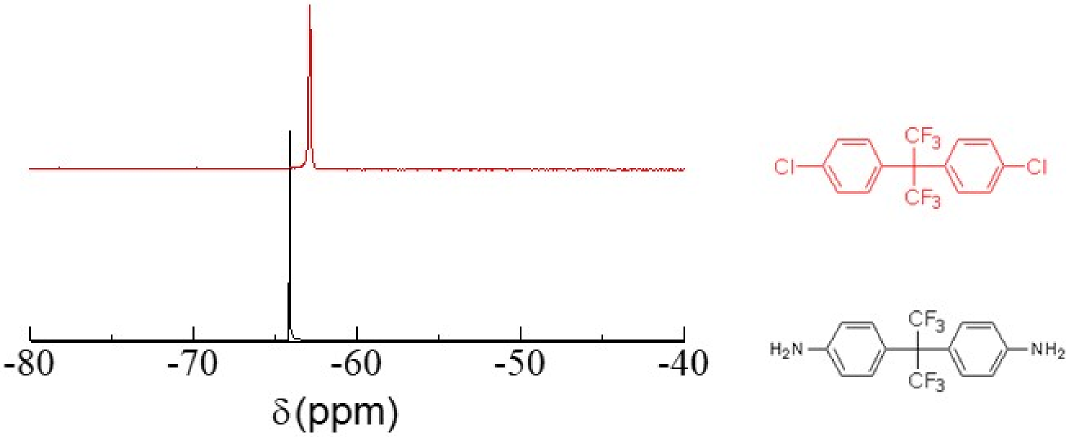

Fig. S3 ^{19}F NMR spectra of 2,2-bis(4-chlorophenyl)hexafluoropropane (**1**) (top) and 2,2-bis(4-aminophenyl)hexafluoropropane (bottom) in $CDCl_3$.

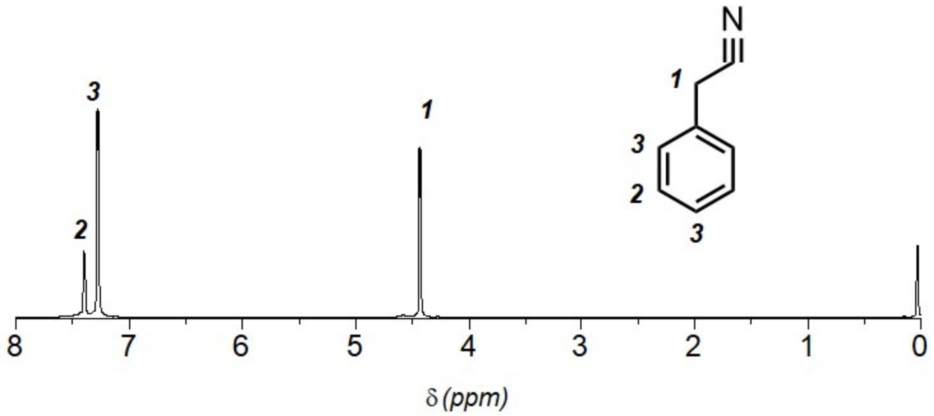

Fig. S4 ^{1}H NMR spectrum of phenylacetonitrile in $CDCl_3$.

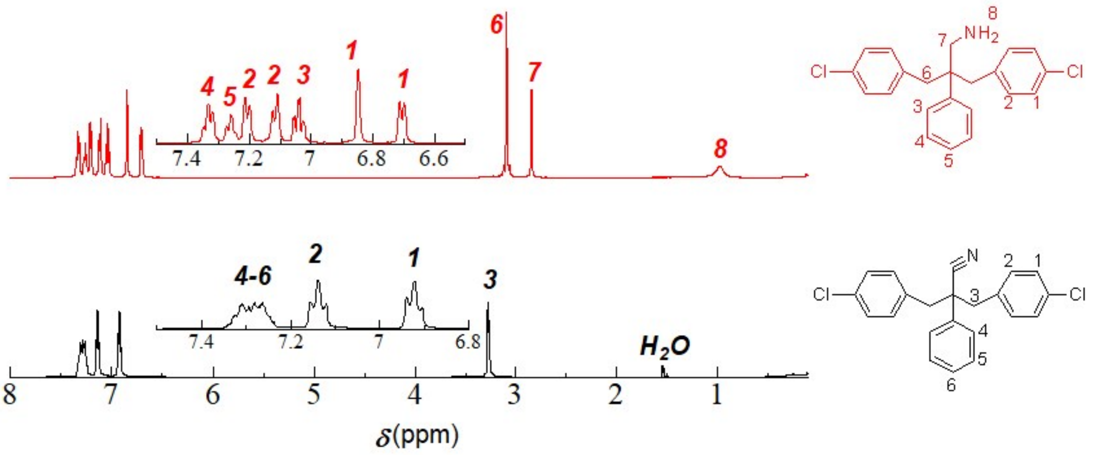

Fig. S5 ^{1}H NMR spectra of 2,2-bis(4-chlorobenzyl)-2-phenyl-ethylamine (**3**) (top) and 2,2-bis(4-chlorobenzyl)-2-phenylacetonirtile (**2**) (bottom) in $CDCl_3$.

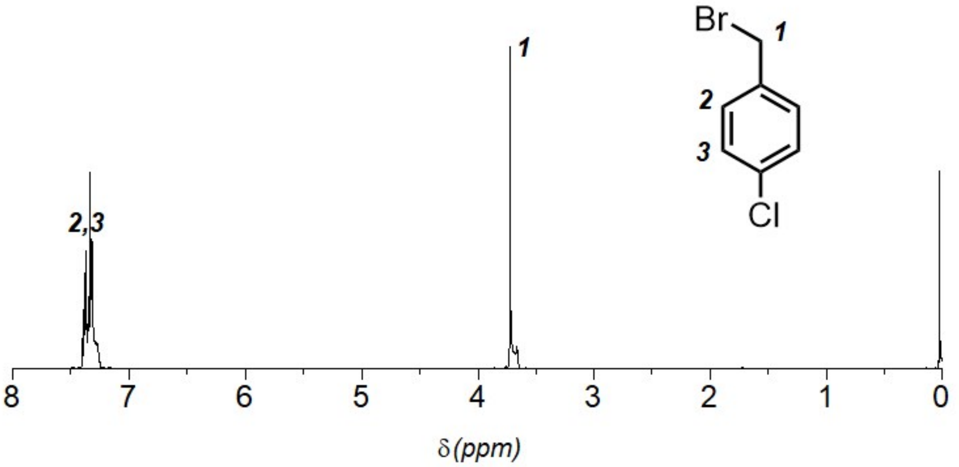

Fig. S6 ^{1}H NMR spectrum of 4-chlorobenzyl bromide in $CDCl_3$.

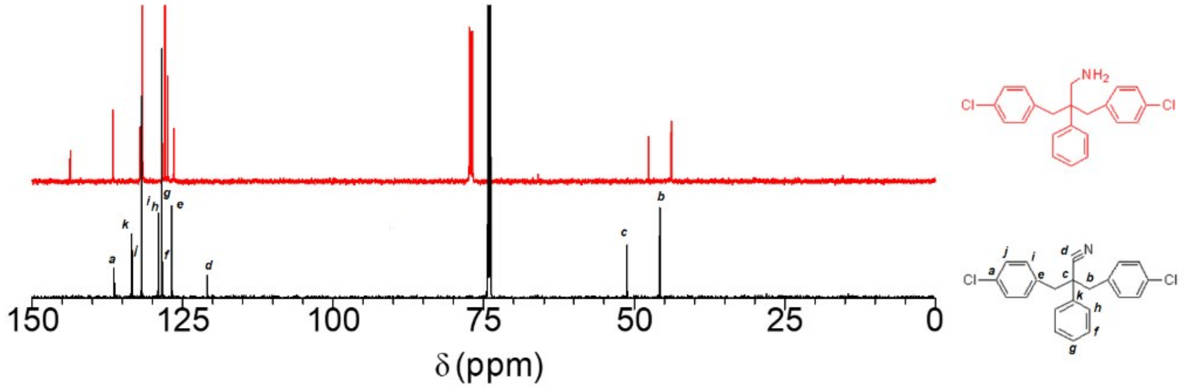

Fig. S7 ^{13}C NMR spectra of 2,2-bis(4-chlorobenzyl)-2-phenyl-ethylamine (**3**) (top) and 2,2-bis(4-chlorobenzyl)-2-phenylacetonirtile (**2**) (bottom) in $CDCl_3$.

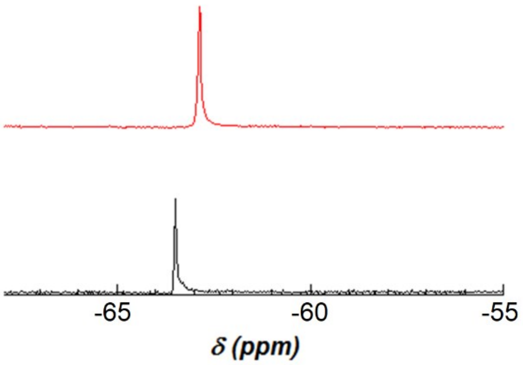

Fig. S8 ^{19}F NMR spectra of QBAF-BS in DMSO-d_6 (top) and BAF-BS in $CDCl_3$ (bottom).

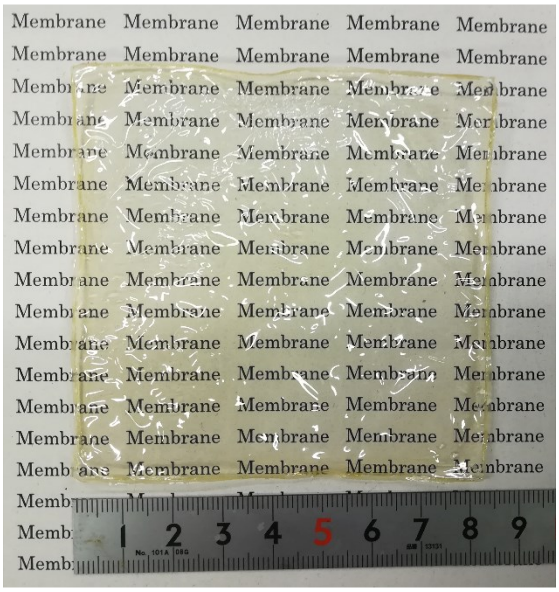

Fig. S9 QBAF-BS2.25 membrane (thickness = 40 ± 7 μm) obtained via casting from DMSO solution at 50 °C overnight.

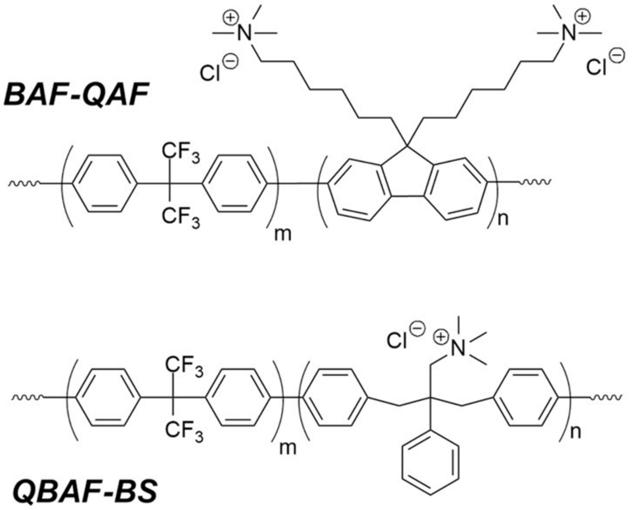

Fig. S10 Structure of BAF-QAF and QBAF-BS copolymers.

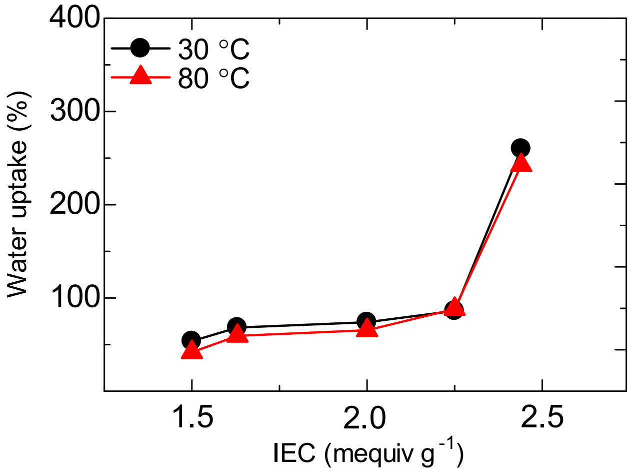

Fig. S11 Water uptake (at 30 and 80 °C) of QBAF-BS membranes as a function of ion exchange capacity.

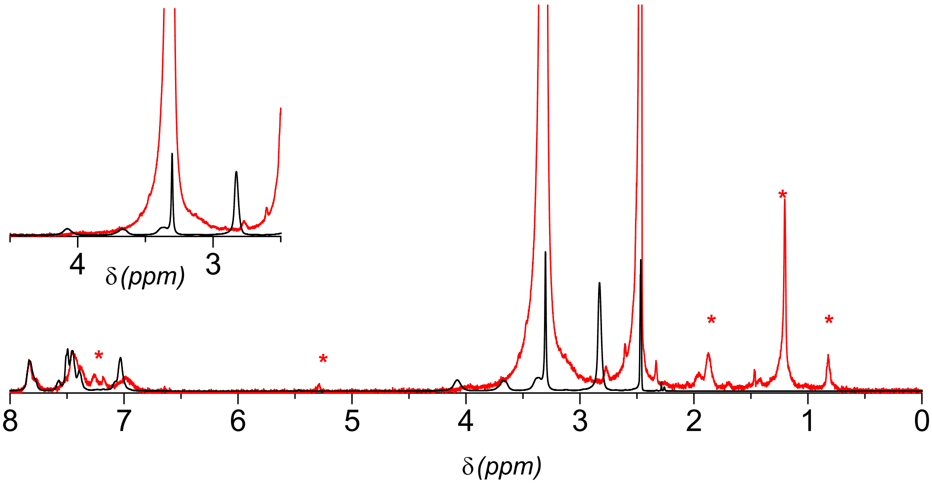

Fig. S12 ^{1}H NMR spectra of the pristine (black, 0 h) and post-test (red, 150 h) QBAF-BS2.00 membrane.

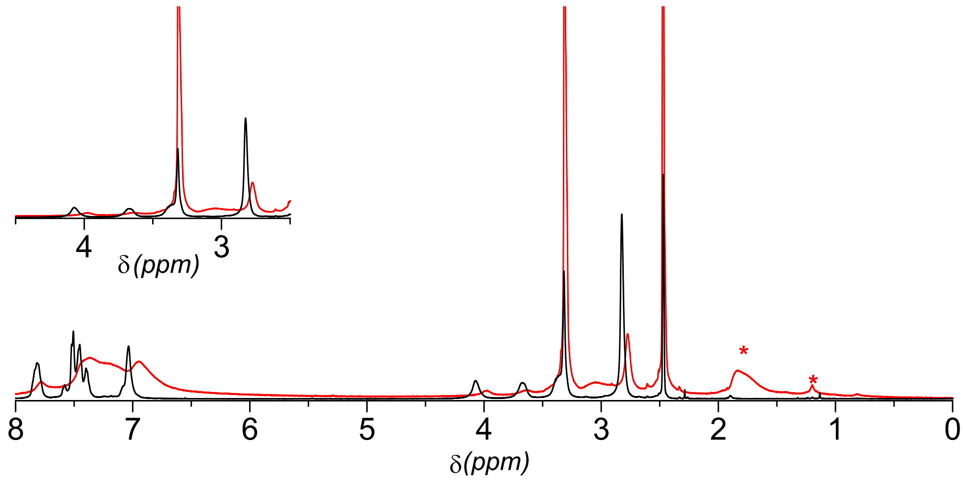

Fig. S13 ^{1}H NMR spectra of the pristine (black, 0 h) and post-test (red, 300 h) QBAF-BS2.25 membrane.

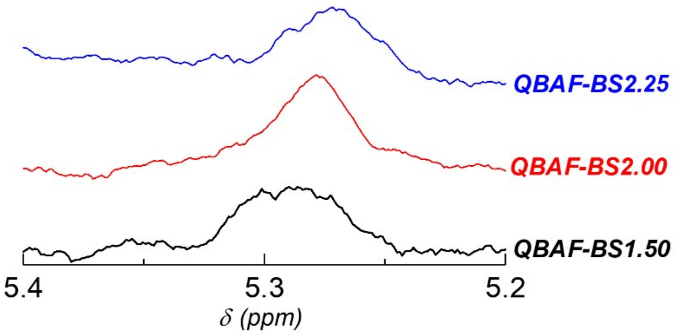

Fig. S14 ^{1}H NMR spectra of QBAF-BS copolymers after the alkaline stability test.

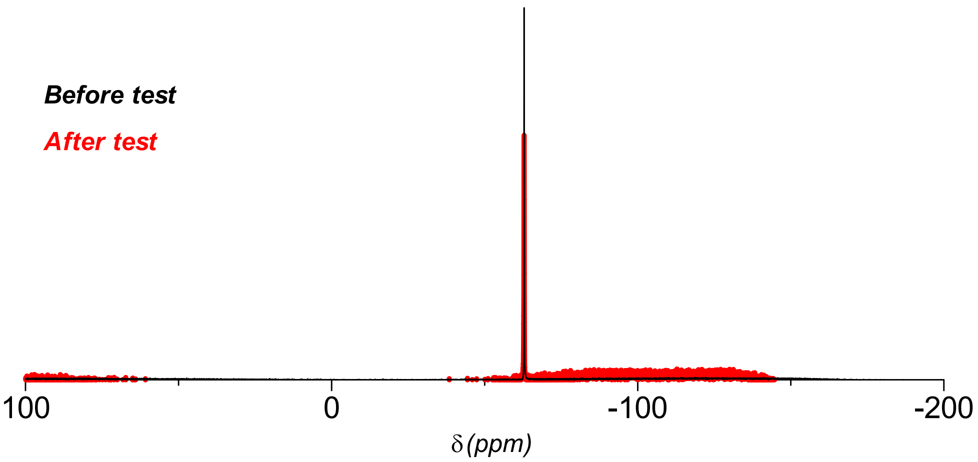

Fig. S15 ^{19}F NMR spectra of QBAF-BS1.50 before (black, 0 h) and after (red, 300 h) the alkaline stability test.

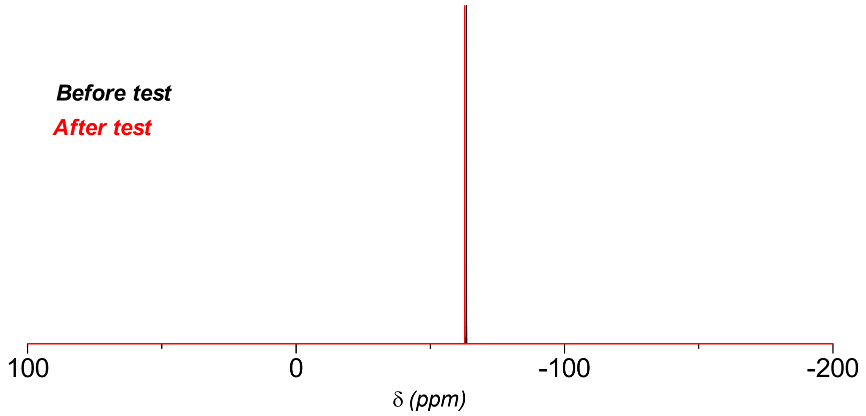

Fig. S16 ^{19}F NMR spectra of QBAF-BS2.00 before (black, 0 h) and after (red, 150 h) the alkaline stability test.

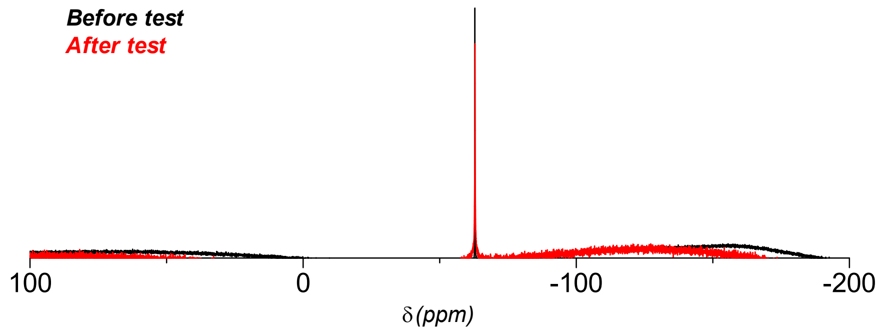

Fig. S17 ^{19}F NMR spectra of QBAF-BS2.25 before (black, 0 h) and after (red, 300 h) the alkaline stability test.

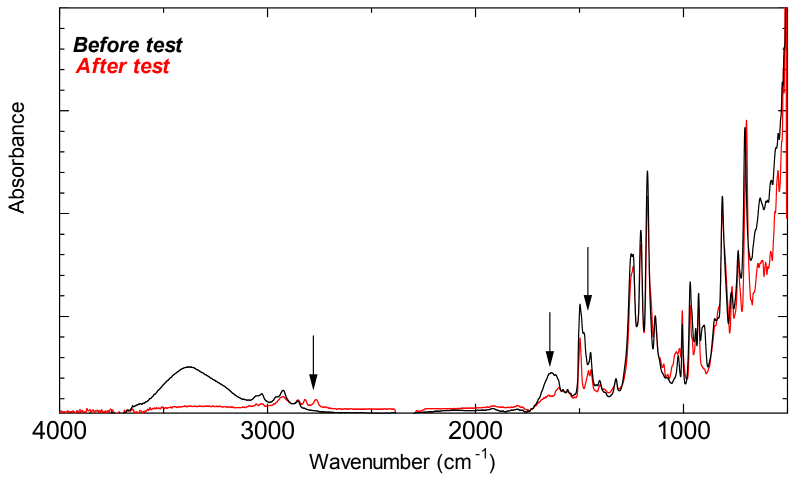

Fig. S18 FTIR spectra of the pristine (black, 0 h) and post-test (red, 150 h) QBAF-BS2.00 membrane.

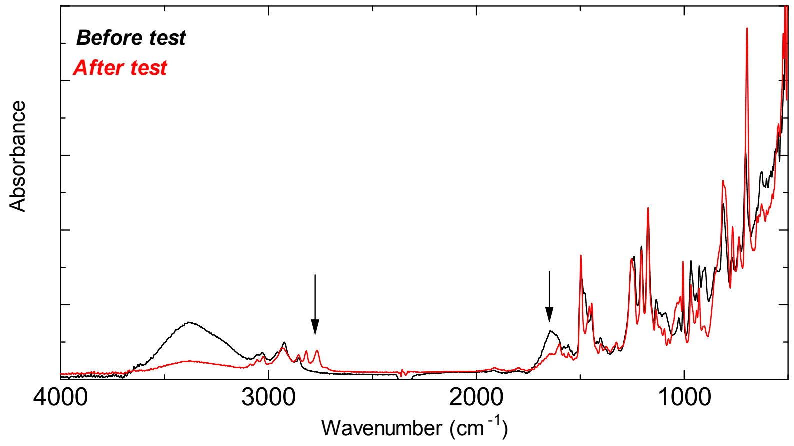

Fig. S19 FTIR spectra of the pristine (black, 0 h) and post-test (red, 300 h) QBAF-BS2.25 membrane.

Reference

1 D. Koronka, A. M. A. Mahmoud and K. Miyatake, *J. Polym. Sci., A: Polym. Chem.*, 2019, **57**, 1059-1069.
